# Maturity Framework for Operationalizing Machine Learning Applications in Health Care: Scoping Review

**DOI:** 10.2196/66559

**Published:** 2025-09-19

**Authors:** Yutong Li, Julie Tian, Ariana Xu, Russell Greiner, Jake Hayward, Andrew James Greenshaw, Bo Cao

**Affiliations:** 1 Department of Psychiatry, University of Alberta, 4-142 KATZ, Edmonton, AB, T6G 2R3, Canada, 1 780-407-6504; 2 Department of Computer Science, University of Alberta, Edmonton, AB, Canada; 3 Department of Emergency Medicine, University of Alberta, Edmonton, AB, Canada

**Keywords:** maturity framework, health care applications, scoping review, machine learning, ML, machine learning operations, MLOps, data, database, clinical practice, health care, health care implementations

## Abstract

**Background:**

The exponential growth of publications regarding the application of machine learning (ML) tools in medicine highlights the significant potential for ML to revolutionize the field. Despite the multitude of literature surrounding this topic, there are limited publications addressing the implementation and feasibility of ML models in clinical practice. Currently, Machine Learning Operations (MLOps), a set of practices designed to deploy and maintain ML models in production, is used in various information technology and industrial settings. However, the MLOps pipeline is not well researched in medical settings, where multiple barriers exist to implementing ML pipelines into practice.

**Objective:**

This study aims to detail how MLOps is implemented in health care and propose a maturity framework for the health care implementations.

**Methods:**

A scoping review search was conducted according to the Joanna Briggs Institute Manual for Evidence Synthesis. Results were synthesized using the 3-stage basic qualitative content analysis. We searched 4 databases (eg, MEDLINE, Embase, Web of Science, and Scopus) to include any studies that involved proof of concept or real-world implementation of MLOps in health care. Studies not reported in English were excluded.

**Results:**

A total of 19 studies were included in this scoping review. The MLOps workflow identified within the studies included (1) data extraction (19/19 studies), (2) data preparation and engineering (18/19 studies), (3) model training (19/19 studies), (4) measured ML metrics and model evaluation (17/19 studies), (5) model validation and test in production (14/19 studies), (6) model serving and deployment (15/19 studies), (7) continuous monitoring (14/19 studies), and (8) continual learning (13/19 studies). We proposed a 3-stage MLOps maturity framework for health care based on existing studies in the field, that is, low (5/19 studies), partial (1/19 studies), and full maturity (13/19 studies). There were 8/19 studies that discussed ethical, legislative, and stakeholder considerations for MLOps implementations in health care settings.

**Conclusions:**

We investigated the implementation of MLOps in health care with a corresponding maturity framework. It is evident that only a limited number of studies reported the implementation of ML in health care contexts. Hence, it is imperative that we shift our focus toward creating an environment that supports the development of ML health care applications, such as improving existing data infrastructure, and engaging partners to support the development of MLOps applications. Specifically, we can include patients, policymakers, and health care professionals in the creation and implementation of ML applications. One of the main limitations includes the varying quality of each extracted study in terms of how the MLOps implementation was presented. Hence, it was difficult to verify the presence and discuss in depth all steps of the MLOps workflow for each study. Furthermore, due to the inherent nature of a scoping review protocol, there may be a compromise on an in-depth discussion of each step within the MLOps workflow.

## Introduction

Over the last decade, there has been exponential growth in the number of machine learning (ML) publications in the medical field, where ML is defined as a group of algorithms and statistical methods that enable computers to learn from data and create predictions [1-3]. Despite the significant number of such studies, fewer than 10% have been implemented in clinical settings [4]. ML model implementations in the clinical setting have significant potential to improve patient care, as ML models have been shown to be effective in medical use cases, such as classifying images, supporting medical diagnoses, and triaging patients [5-10]. To best operationalize the ML models, a general standardized workflow can help enable the universal implementation of ML systems across multiple health care disciplines and sites to ensure that ML models are being implemented in an ethical and practical manner. The ethical and practical implementation of Machine Learning Operations (MLOps) allows for the continued implementation of the ML tool in clinical practice while improving patient care [11,12]. Because the MLOps principles are a pre-existing framework that is adopted successfully in many industrial fields, such as technology, finance, and transportation, it may be a promising avenue to establish an MLOps workflow that can be standardized to ensure universal quality of ML tools across multiple medical domains and sites [11]. Here, we investigate how MLOps is adopted in the health care field, including considerations for implementing MLOps and propose a maturity framework within the context of health care applications.

MLOps is an extension of development operations, a software engineering practice that involves continuous integration (CI) and continuous delivery of software to ensure speed and high quality of production [11,13]. MLOps incorporates frameworks, such as continual learning (CL), where models are retrained and deployed based on new data when there is a decay in model performance; and continuous monitoring (CM) where the performance of the ML model is monitored [11,14]. Currently, the standards for MLOps include variations of the following steps: data preparation (data extraction and data engineering); model development (model training and measuring model performance); and model operationalization (model validation and testing in production, model serving and deployment, CM and CL) [11,12,14,15]. The first stage involves defining the use case and outcomes to inform the data collection and handling process. Data and feature engineering follows the data collection process, where important features or predictors are created. Furthermore, the data will be cleaned and normalized according to the requirements of the use case and model [12]. Following this stage, the best ML algorithm and hyperparameters will be selected using ML performance metrics. When the ML model is assessed to be the best model, the model will be presented to end-users on a user-friendly platform [16]. MLOps is the presence of CM, where the ML model performance is continuously monitored, and when the above steps are automated, the ML model is updated on new data when the ML model decays to a predefined metric (CL), allowing for the preservation of model performance [14].

Despite the emphasis on CM and CL within MLOps, not all organizations or use cases have the capability to reach full MLOps maturity [11,17]. There are various descriptions for the stages of MLOps maturity, with varying degrees of the implementation of CM and CL in the MLOps framework [11,17-20]. For example, Google has outlined the three stages of maturity for presented MLOps processes: (1) Manual process, (2) ML pipeline automation, and (3) CI and continuous delivery automation [19]. For the first stage, companies perform every step of the MLOps workflow pipeline manually. A problem with this method is that the ML framework is not frequently updated or monitored. The second stage is ML pipeline automation, where the data ingestion and data engineering steps are automated, allowing for the automation of model retraining and testing to ensure continuous delivery of model predictions. However, the deployment of the ML pipeline is completed manually. The final stage of ML pipeline automation is where the data ingestion and engineering steps, model training, testing, validation, serving, monitoring, and deployment of the ML pipeline are all automated. Microsoft has outlined a 5-level model, where MLOps practices are absent at level 0 as the ML model training and deployment are manually completed. Level 4, or full MLOps automated operations, is defined as the automated training, monitoring, and deployment of an ML system. Similar to the Microsoft maturity model, Garg et al [18] have outlined a 3-stage model where level 0 is the absence of automation of the ML system and level 2 is the full maturity of the system. John et al [11] described an MLOps maturity model from a different perspective, where the first stage of the maturity model is when an organization adopts automated data collection, and the final stage is when the organization adopts automated data collection, model deployment, and model monitoring.

In the context of health care, there are distinct considerations for the application of MLOps, as health care presents unique challenges to implementing MLOps. For instance, the clinical environment is unique in that we have additional stakeholder considerations (eg, clinician, patient, and community considerations), regulatory considerations, and considerations surrounding health care data [21]. Because of the evolving nature of clinical practice, regulatory environment, and population dynamics, there is a benefit to implementing a fully mature MLOps pipeline workflow with the inclusion of human oversight [22-25]. This is because the ML model’s performance may decay as time progresses due to shifts in the patient population in terms of the patient demographics and the operation of the clinical environment (eg, changes in medical technologies, new procedures, and emergence of new disease outbreaks), which highlights the potential added benefit of an adaptive ML prediction system.

In this paper, we will investigate how the MLOps framework has been applied to health care, considerations for implementing MLOps, and the maturity framework within the context of health care applications. For our purposes, we have chosen to adopt a hybrid of all the MLOps maturity models for health care implementations, which can be applied to the literature described in the scoping review. In the hybrid description of the maturity model, we have identified 3 stages of maturity within the studies selected for this scoping review. These 3 stages include low, partial, and full maturity. Low maturity is the complete absence of CM and CL. Partial maturity is the presence of CM, with a lack of CL, as the retraining of the model was manually triggered by the engineering team. Full maturity is the presence of CM and CL.

## Methods

### Search Strategy

The Joanna Briggs Institute framework for scoping reviews was used to guide the execution of this study, as this framework provides a structured approach for searching, charting, and analyzing the data [26-31]. Details regarding the scoping review framework are presented in the PRISMA-ScR (Preferred Reporting Items for Systematic Reviews and Meta-Analyses extension for Scoping Reviews), a checklist of essential items for scoping review reporting (Checklist 1) [32]. We conducted a 3-step search strategy proposed by Aromataris and Riitano [33]. The first 2 steps involved 2 rounds of searching, which were conducted to ensure that the search process was comprehensive. The first round consisted of a search of 4 databases (MEDLINE, Web of Science, Scopus, and Embase) and subsequent analysis of relevant keywords in the title and abstract of extracted literature (Multimedia Appendix 1). We have chosen these 4 databases because they capture the medical, engineering, and computer science fields. To specify, studies were screened for title, abstract, and keywords by reviewers YL and JT, with AX resolving conflicts. We collected keywords relating to MLOps and health care that were not included in the first round of searching. These keywords were incorporated into the final search terms described in Multimedia Appendix 1, and a second search where a total of 2712 studies were identified. The second search expanded the initial search terms to include a broader range of studies. The third step involved searching through the reference list of selected studies to identify additional papers, which were added to the papers that were screened. Five studies were identified and manually uploaded following hand-searching the reference lists of included studies. The most recent search was conducted on 29 January 2025.

### Search Terms

Search terms relating to ML, development operations, and all health care fields were included. To ensure that search terms were comprehensive, 2 rounds of searching were performed. The first round involved collecting and brainstorming search terms relating to ML, MLOps, and health care. The second search involved collecting search terms from the first round of abstract, title, and keyword screening for the studies collected. For example, the term MLHOps was not present in the search terms for the first round of searching. However, it was identified as a keyword in one study within the first round of the search; hence, it was incorporated as a search term for the second round of searching. Finalized search terms from the Web of Science, Embase, Ovid MEDLINE, and Scopus were presented in Multimedia Appendix 1. Finally, we hand-searched the reference lists of the included studies for other potential MLOps studies that were not identified.

### Selection Criteria

We used the PCC framework (“Population,” “Concept,” and “Context”) to select the studies that will be included in the scoping review (Table 1; [31]). Population was defined as any population, as MLOps can be applied to any individual in any country in a health care setting. The main concept of interest is MLOps. The context pertains to all health care practices and fields. We only included studies where MLOps was implemented in a clinical application. This included studies that were a proof of concept of a clinical application, or studies implemented in a real-world clinical setting. Research papers consisting of peer-reviewed and preprint articles that discuss the application of MLOps in health care settings were included in this review. Studies were excluded if they were not written in English, did not discuss an instance of MLOps implementation in a medical setting, or did not focus on health care applications. Perspective pieces describing MLOps concepts were also excluded. There were no set limitations on the publication date.

**Table 1.** Summary of the “population,” “concept,” and “context” framework used in this scoping review, exclusion criteria, and other additional inclusion criteria.

Category	Description
Population	Any population with a medical condition.
Concept	Any applications or proof of concept involving MLOps^a^.
Context	All clinical practices and fields.
Inclusion criteria	Research articles consisting of peer-reviewed and preprint articles that discuss the application of MLOps in health care settings.
Exclusion criteria	Studies were excluded if they were not written in English, did not discuss an instance of MLOps implementation in a medical setting, or did not focus on health care applications. Perspective pieces describing MLOps concepts were also excluded.

a MLOps: Machine Learning Operations.

### Study Selection

Following the search for the studies, identified studies were first exported into Zotero (Corporation for Digital Scholarship) and then imported into Covidence (Veritas Health Innovation Ltd), an online platform for managing reviews. Duplicates were removed before title and abstract screening. Title and abstract screening and full-text screening were completed separately by YL, JT, and AX, and each study was reviewed by 2 reviewers according to the PCC criteria mentioned in the “search criteria” section. Conflicts that occurred in either stage were resolved by consensus among the reviewers.

### Data Charting and Synthesis

The data extraction template was developed by YL using the 3-stage basic qualitative content analysis discussed by Pollock et al [31], and Elo and Kyngäs [34]. For the organization stage, the deductive approach was used, where an extraction framework was developed using pre-existing MLOps frameworks in literature [11,12,14,15,17,19,20]. For the MLOps maturity model, we adopted an inductive approach to account for the various interpretations of the MLOps maturity framework found in the literature. YL, JT, and AX analyzed the 19 chosen studies to find a common MLOps maturity framework that accommodates the diverse perspectives presented in these works [34-52]. Extraction was performed by YL and JT, with AX resolving any conflicts within the Covidence platform. The extraction form included the author and year, aim of the study, population and disease characteristics, location of study, MLOps maturity stage, MLOps workflow (data extraction, data preparation and data engineering, model training, measured ML metrics and evaluation, model validation and test in production, model serving and deployment, and CM and CL), and other considerations for MLOps in health care applications. The data from the extraction template were organized into Table 2 (author and year, aim of the study, population and disease characteristics, and location of study), Table 3 (MLOps workflow), and Table 4 (other considerations for MLOps in health care applications) (Multimedia Appendix 2).

**Table 2.** The objectives of each study included in the scoping review, along with the year of publication, author(s), study aim, study location, and the population or disease characteristics of individuals in the dataset used for Machine Learning Operations (if applicable).

Author and Year of publication	Population and Disease characteristics	Location	Aim of study
Bahaa et al [35], 2023	Patients with heart disease	United States	This study introduces a proof-of-concept model, aiming to turn unprocessed data into a useful product that provides utility through a rapid, scalable, and repeatable process. The proof-of-concept model was demonstrated through the UCI (University of California Irvine) heart dataset.
Bai et al [36], 2022	Emergency Department Visits	United States	This study aimed to develop a prototype Machine Learning Operations (MLOps) platform for machine learning (ML)–based clinical tools.
Granlund et al [37], 2021	Patients undergoing joint replacement surgery	Finland	This study presents a case study of Oravizio (Solita), a ML-based medical device used for predicting the risk of joint replacement surgery, in addition to discussing policy considerations for ML-based medical devices.
Kanbar et al [38], 2022	Patients diagnosed with epilepsy	United States	This study seeks to develop clinical decision support tools that integrate electronic health records and patient notes to make predictions on surgical candidacy for patients with seizure and to screen emergency department patients for eligibility to enroll in clinical trials.
Karácsony et al [39], 2021	Patients diagnosed with epilepsy	Germany	In this study, an MLOps framework was tailored to the analysis of 2D and 3D seizure videos to classify different types of seizures.
Kleftakis et al [40], 2022	N/A^c^	Greece	This study presents an MLOps framework that monitors the health of the entire body and the patient’s overall health status.
Krishnan et al [41], 2022	Internal medicine, critical care, and patients with chest X-rays	Canada	This study details the MLOps framework applied to several medical use cases, such as mortality prediction and imaging applications.
Kundu and Bilgaiyan [42], 2023	Patients diagnosed with COVID-19	India	In this study, a different and efficient approach to automating the hyper-parameter tuning process is proposed with the use of DevOps^a^ tools for automating the workflow.
Meel and Bodepudi [43], 2021	Patients with skin cancer	United States	This study presents Melatect, a ML prediction tool embedded in an IOS app that predicts the malignancy of a skin lesion.
Mirza et al [44], 2023	N/A	United States	This study details the development of an MLOps platform to predict the workload at clinical trial sites.
Tougui et al [45], 2022	Patients diagnosed with Parkinson	Morocco	This study presents a ML application that uses voice recordings to predict the presence of Parkinson disease. The prediction is presented in a web application.
Tseng et al [46], 2022	Patients undergoing cardiac arrest	Taiwan	This study defined a new implementation guide for running a user-friendly ML system for predicting In-hospital cardiac arrest (IHCA). This system uses the Fast Healthcare Interoperability Resources (FHIR) to manage health care data and the ML application.
Ghosh and Chaki [47], 2025	Patients with kidney cancer	India	This study aims to automate the detection of kidney tumors from computed tomography scans via the integration of MLOps principles for generating predictions.
Imrie et al [48], 2023	Patients with Diabetes	United States	This study seeks to automate the training and deployment of ML models in clinical environments to support physician decision-making, with a demonstration in diabetes risk prediction.
Lombardo et al [49], 2024	Hospital staff and patients	Italy	This study provides a proof-of-concept model for the location-based service tracking of hospital staff to make predictions on the location-based trajectories of hospital staff. This has potential use cases in the prediction of the trajectories of the hospital staff to determine potential wait times in the hospital or to identify potential security concerns.
Lutnick et al [50], 2023	N/A	United States	This study aims to develop an MLOps-based system that enables nontechnical users, such as clinicians, to leverage pretrained ML models to develop predictions for histology images.
Markowitz et al [51], 2024	Patients with central nervous system tumors	United States	This study aims to develop an MLOps pipeline for classifying central nervous system tumors using data from clinical diagnostic tools, including the MethylationEPIC v2.0 BeadChip (Illumina) and other DNA/RNA biomarker-based cancer screening technologies.
Mathew and Joseph [52], 2023	Patients with brain cancer	India	This study develops an MLOps pipeline for the prediction of brain tumors using MRI^b^ images.
Moskalenko and Kharchenko [53], 2024	N/A	Ukraine	This study aims to develop a resilient MLOps system for health care applications, as systems in health care applications may be impacted from adversarial attacks and distribution drifts.

a DevOps: Development Operations

b MRI: Magnetic Resonance imaging.

c N/A: not applicable.

**Table 3.** The checklist for Machine Learning Operations pipeline and Machine Learning Operations maturity^a^.

Author and year of publication	MLOps^b^ maturity level	Data preparation		Model development		Model operationalization		
		Data extraction	Data engineering	Model training	Measured ML^c^ Metrics and Evaluation	Model validation and test in production	Model serving and deployment	Continuous (CM^d^, CL^e^)
Granlund et al [37], 2021	Low maturity	✓^f^	✓	✓	✓	✓	✓	
Karacsony et al [39], 2021	Low maturity	✓	✓	✓			✓	
Tougui et al [45], 2022	Low maturity	✓	✓	✓	✓	✓	✓	
Imrie et al [48], 2023	Low maturity	✓	✓	✓	✓	✓	✓	
Lutnick et al [50], 2023	Low maturity	✓	✓	✓		✓	✓	
Bahaa et al [35], 2023	Partial maturity	✓	✓	✓	✓		✓	CM only
Tseng et al [46], 2022	Full maturity	✓	✓	✓	✓		✓	✓
Kanbar et al [38], 2022	Full maturity	✓	✓	✓	✓	✓	✓	✓
Kleftakis et al [40], 2022	Full maturity	✓	✓	✓	✓	✓	✓	✓
Krishnan et al [41], 2022	Full maturity	✓	✓	✓	✓			✓
Kundu and Bilgaiyan [42], 2023	Full maturity	✓	✓	✓	✓			✓
Meel and Bodepudi [43], 2021	Full maturity	✓	✓	✓	✓	✓	✓	✓
Mirza et al [44], 2023	Full maturity	✓	✓	✓	✓	✓	✓	✓
Bai et al [36], 2022	Full maturity	✓	✓	✓	✓	✓	✓	✓
Ghosh and Chaki [47], 2025	Full maturity	✓	✓	✓	✓	✓	✓	✓
Lombardo et al [49], 2024	Full maturity	✓	✓	✓	✓	✓	✓	✓
Markowitz et al [51], 2024	Full maturity	✓	✓	✓	✓	✓		✓
Mathew and Joseph [52], 2023	Full maturity	✓	✓	✓	✓	✓	✓	✓
Moskalenko and Kharchenko [53], 2024	Full maturity	✓		✓	✓	✓		✓

a Blank cells denote that a MLOps step was not discussed or found within the paper.

b MLOps: Machine Learning Operations.

c ML: Machine Learning.

d CM: Continuous Monitoring.

e CL: Continual learning.

f Presence of the MLOps framework step.

**Table 4.** Other health care–specific considerations for the Machine Learning Operations framework.

Author and year of publication	Other considerations
Bahaa et al [35], 2023	Team considerations for building the DataOps^a^ pipeline were discussed as team members relating to domain knowledge and technical skill sets were considered as crucial aspects of the pipeline. Other considerations include the archiving or deleting of unused data to ensure the pipeline is cost-effective.
Bai et al [36], 2022	The authors outlined the core tenets of an MLOps^b^ system in health care, which are that the platform must be ethical, auditable, adaptable, automated, and accessible. This ensures that patient privacy is protected, the machine learning (ML) system is transparent, and it maintains a safe level of performance. Team considerations were key stakeholders like a health informatics professional, health care researcher, and chief information officer that work together to define the key clinical use case and govern the development and safety of the ML model.
Granlund et al [37], 2021	This paper also focused on the regulatory considerations for ML-based medical device systems. Four important considerations for deploying ML-based medical device systems in clinical practice involve the medical device’s ability to provide clinical benefit to the target patient group, transparency of the device, the performance or safety of the medical device, and risk management of the ML system.
Kanbar et al [38], 2022	Considerations for patient privacy were discussed as the servers used for prediction ran on a secured Health Insurance Portability and Accountability Act-compliant server. Team considerations for building an MLOps pipeline. Need to consider the team structure. Clinicians, researchers, support health care staff, and research coordinators worked with the bioinformaticians to create this health care framework.
Karácsony et al [39], 2021	Considerations for data storage take into account the health data privacy laws in the European Union. Due to data privacy concerns, the epilepsy data is stored in a centralized private server in the cloud where the data can only be accessed via the encrypted Virtual Private Network connection. Patient identifiers were also removed to maintain privacy. There is also consideration of computational speed and resources in the creation of the MLOps pipeline highlighted in this paper as a central server that hosts the patient data and ML models to minimize the transfer of large file sizes.
Imrie et al [48], 2023	This study also details the importance of defining the advantages of using ML over traditional statistical methods for clinical prediction and demonstrating the importance of feature importance for debugging ML models within the production pipeline.
Lutnick et al [50], 2024	Considerations for data access were discussed and integrated into the MLOps platform for histology image analysis.
Moskalenko and Kharchenko [53], 2024	In medical applications, there is more need for MLOps systems to be more resilient as it is a high-stakes environment. Hence, MLOps systems need to be developed for improved safety and adaptability within the medical system. Considerations for data protection and model development were discussed to be avenues for adapting a MLOps system for medical applications.

a DataOps: Data Operations.

b MLOps: Machine Learning Operations.

## Results

### Data Analysis and Presentation

**Figure 1.**
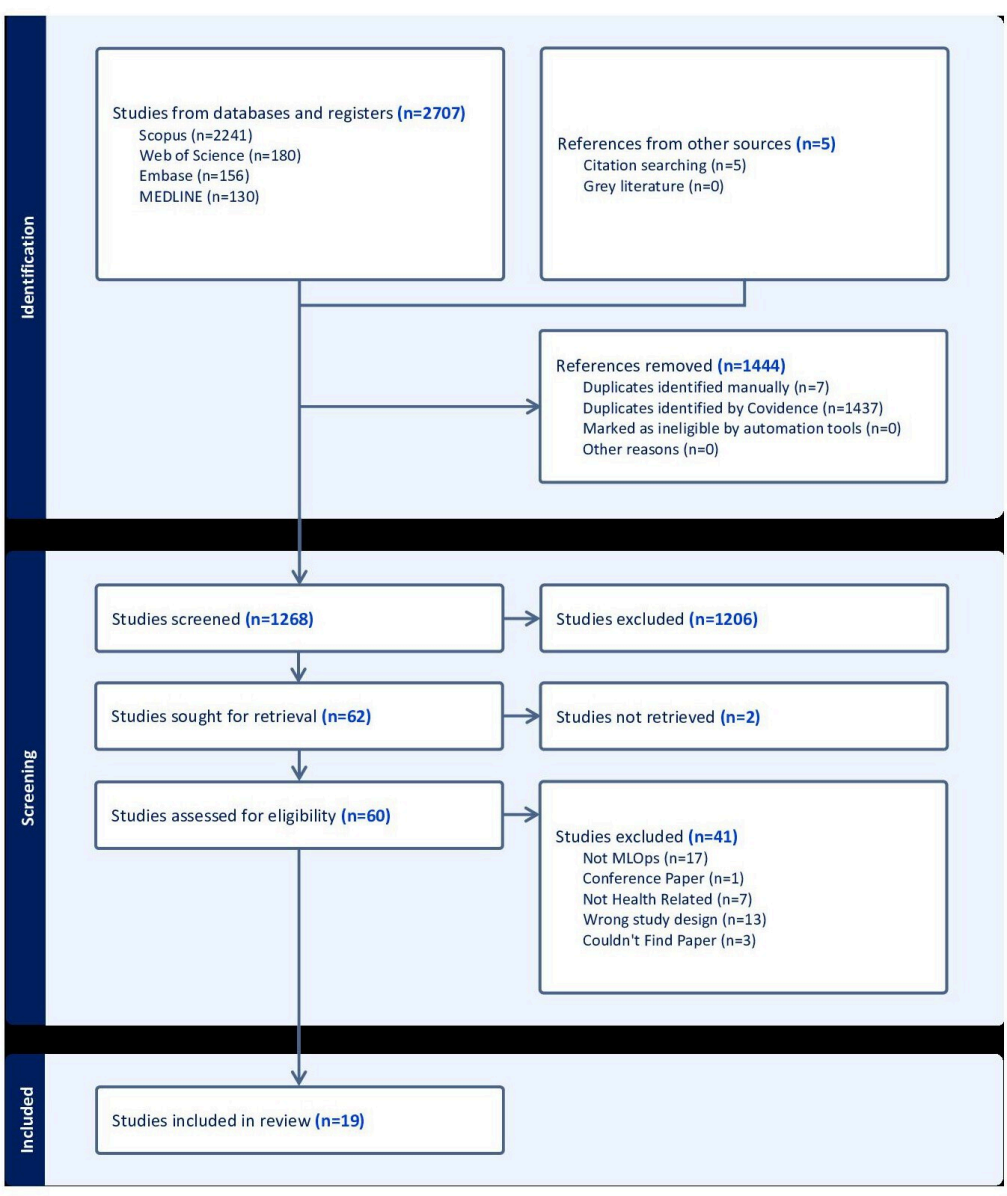
The PRISMA (Preferred Reporting Items for Systematic Reviews and Meta-Analyses) flowchart for screening Machine Learning Operations health care studies. MLOps: Machine Learning Operations.

**Figure 2.**
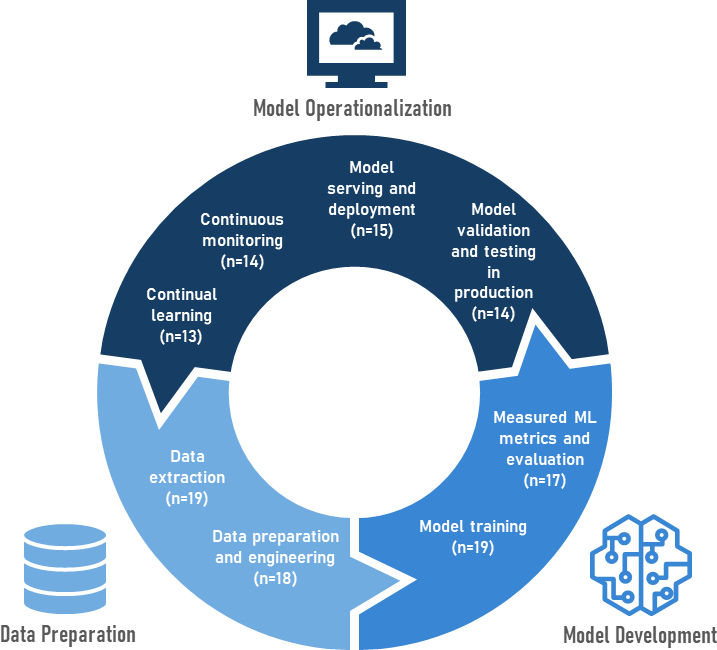
Graphical representation of Machine Learning Operations workflow pipeline and maturity stage. ML: machine learning.

The search protocol was described as a PRISMA (Preferred Reporting Items for Systematic Reviews and Meta-Analyses) flowchart in Figure 1. In total, 2712 studies were identified based on the search terms in Multimedia Appendix 1 from Scopus, Web of Science, Embase, Ovid MEDLINE, and the reference list of included papers. In the screening phase, 1268 studies were screened following the removal of 1444 duplicate studies. There were 1206 excluded studies based on the title, abstract, and keyword screening. Papers without MLOps and health care–related terms or concepts in the title, abstract, and keyword screening were excluded. A full-text screening was completed for the 62 studies to assess eligibility for inclusion. The final 19 extracted papers were presented in 3 tables. Table 2 describes the aims of each selected study, year of publication, author, location where the study was conducted, and the population and disease characteristics. Table 3 describes elements of the MLOps workflow pipeline used by each of the studies, in addition to a summary of the MLOps maturity model for each study. Table 4 outlines additional considerations for health care applications not present within the MLOps workflow pipeline. Figure 2 outlines the MLOps workflow pipeline as a graphical representation of the number of studies per stage.

### Data Extraction

The MLOps workflow pipeline from all 19 studies discussed the implementation of a data extraction step, where the data used for developing the ML model were extracted from a pre-existing database [35,41-45,47,48,50,52,53], or the collection of data from various electronic health records or the collection of patient physiological measurements from clinical settings [36-40,46,49]. Markowitz et al [51] obtained data from a combination of pre-existing databases and in-hospital measurements.

### Data Preparation and Data Engineering

Data preparation and engineering involve loading data into a database, preprocessing raw data to create relevant predictive variables or features, and cleaning and formatting data so it is suitable for training and testing. There were 18 studies [35-52] that described data preparation and engineering steps for deploying the ML model. Because medical applications require the protection of patient privacy, studies such as Bai et al [36] described the removal of 18 protected health information identifiers. Kanbar et al [38], and Lutnick et al [50], also discussed the importance of protecting sensitive patient data during the MLOps workflow. To select the top features for model training, algorithms such as least absolute shrinkage and selection operator, or ElasticNet were used on the training dataset [37,45]. The assessment of data quality was also discussed in Kleftakis et al [40]. Moskalenko and Kharchenko [53] did not describe the feature engineering step involved in the creation of their MLOps workflow.

### Model Training

The model training step was described in all 19 studies [35-53] for the development of the models. Studies also emphasized the importance of transparency and of the ML model within the context of health care applications [37].

### ML Model Evaluation

There were 17 studies [35-38,40-49,51-53] out of 19 studies that described the model metrics used to evaluate the ML models used for the studies. Examples of metrics used to evaluate model performance include accuracy, F1 (harmonic mean of precision and recall), the area under the receiver operating curve, sensitivity, and specificity. Two studies [39,50] did not describe the ML model evaluation technique used.

### Model Validation and Testing in Production

Model validation and testing in production involve elements relating to internal and external validation. Internal validation involves assessing the performance of the ML model on the dataset used to develop the model [54]. External validation involves the assessment of the ML model performance on a dataset not used for the development of the ML model. Furthermore, testing in production refers to the process of testing the model in a real-world clinical scenario. Examples of testing in production may refer to the comparison of the model prediction to a clinician’s prediction. Fourteen [34,36-38,41,42,45-52] out of 19 studies discussed how the ML models were validated and tested in production. A total of 11 studies [37,38,40,44,45,47-50,52,53] performed internal validation only and 3 studies [36,43,51] performed both external and internal validation.

There were 2 studies that emphasized the importance of testing the model in production to ensure the model has clinical benefits and is ethical for use in patient populations. Kanbar et al [38] performed shadow deployment, which is the comparison of ML model accuracy to the clinician’s accuracy in terms of decision-making. Specifically, the prediction from the model was compared against a prediction from an epileptologist for 1 year. In addition to model performance against a baseline, the bias of the model was also considered. Kleftakis et al [40] emphasized the importance of testing whether a model surpassed the accuracy of a previous model before it could be placed into production.

### Model Serving and Deployment

Model serving and deployment is the process of packaging the ML model and releasing it using an application programming interface on a user-friendly interactive platform. Fifteen studies [35-40,43-50,52] out of 19 studies described the process in which the ML model was released into a user-friendly platform.

### Maturity Stage and Continuous Monitoring and Continuous Learning

Here we propose 3 MLOps maturity stages: low maturity, partial maturity, and full maturity. Low maturity was defined as the absence of any CM or CL. Hence, the ML pipeline was used to make predictions with no regard for performance degradation; essentially, it is a “locked” model. Five studies [37,39,45,48,50] out of 19 studies have low MLOps maturity. The second stage is partial maturity, where there was the presence of CM by the MLOps system; however, there was a lack of automatic triggering of the MLOps workflow pipeline, as retraining of the ML was manually scheduled by the engineering team [35]. One study involved partial maturity of the MLOps workflow [35]. The third stage involves the full maturity of the model, where the model is continuously monitored and exhibits CL. Here, the model is retrained using new data from either a pre-existing database or newly collected data, and the updating of the MLOps workflow pipeline is determined by the deterioration in the model’s performance. Thirteen studies [36,38,40-44,46,47,49,51-53] out of 19 studies contained full maturity of their MLOps workflow pipelines.

### Other Considerations Specific to Health Care Applications

Because of the sensitive nature of health care data, considerations relating to ethical practice, privacy, stakeholder management, and MLOps design principles were discussed in 8 studies. Specifically, 5 studies [36-39,50] discussed the importance of data management to protect patient privacy. Two studies [38,39] emphasized the importance of applying server governance to adhere to the legislation present in the region where the study was used. Granlund et al [37] also discussed the importance of legislation on how an MLOps workflow pipeline could be deployed. Notably, the US Food and Drug Administration and the European Union on Medical Devices have supported the use of “locked” or ”frozen” ML models, models that cannot be improved or modified following the release of the model into production [37,55,56]. Another essential aspect of integrating a successful MLOps workflow pipeline into health care applications was the selection of relevant stakeholders and experts [35,36]. Health care professionals and an effective engineering team were needed to run an MLOps workflow pipeline, as health care professionals provide domain knowledge to define the use case and govern the data usage, and the engineering teams work closely with the health care professionals to build the MLOps application to ensure that it is user-friendly. Finally, 2 studies discuss how the MLOps workflow can be adapted to health care applications. Moskalenko and Kharchenko [53] develop a resilient MLOps pipeline that can better withstand adversarial attacks and model performance decays in consideration of the high-stakes environment of health care applications. Imrie et al [48] discuss how an MLOps workflow needs to justify the use of ML in health care applications through the comparison of ML to traditional statistical methods, such as the Cox Proportional Hazards model. Furthermore, the implementation of ML in health care systems should be interpretable to clinical staff for the improved trust in the ML application.

## Discussion

### Principal Findings

MLOps is crucial for health care applications as it provides a structured framework to operationalize ML models, ensuring they are implemented ethically and practically. By following a tailored MLOps framework, health care organizations can streamline the deployment, monitoring, and updating of ML models, thus enhancing patient care and operational efficiency [57]. MLOps also describes a maturity model with various stages, from manual processing of ML pipelines to fully mature pipelines with CM and CL, indicating different stages of maturity and sophistication in the implementation [11,14]. The MLOps workflow and maturity model help organizations understand their current capabilities and identify areas for improvement to achieve higher efficiency and reliability in deploying ML models in health care. In this study, we highlighted the steps of an MLOps workflow pipeline, which included data extraction, data preparation and data engineering, model training, model evaluation, model validation and testing in production, model serving, and CM/CL.

Among all the selected studies, the data extraction phase was present. However, there were differences in the origin of the data among all studies. One major difference was the origin of the data, as 12 studies [35,41-45,47,48,50-53] out of 19 studies used a pre-existing database to test their proof-of-concept MLOps implementation. The fact that the majority of studies use a pre-existing database may suggest that there are barriers to data access, a well-known challenge in digital health tools [58,59]. Especially when these studies originate from diverse locations (Table 2), they suggest that MLOps is still an emerging area of research globally, and it may not be feasible to design large-scale implementation with real-time data in medical applications.

All 19 studies differed in model validation and testing in production, model serving and deployment, and CM and CL [36-39,43-46,48,51,52]. For example, studies differed in how they performed validation. This can be problematic, as the method of validation is a critical step in determining whether an MLOps implementation can be generalized to novel datasets or clinical sites. Internal validation refers to assessing the model’s performance on the dataset used for its development, ensuring it performs well within the known parameters [54]. External validation, however, involves testing the model on a novel dataset, which was not used during the development phase. This step is crucial as it demonstrates the model’s ability to generalize to unseen data, making it the gold standard for validation, as MLOps implementations may need to be used for multiple different scenarios within a clinical domain [60,61]. In addition to validation, models must be tested in the clinical setting to ensure that they can improve patient experience and are able to perform as well or better compared to clinical predictions. Kanbar et al [38] demonstrated an instance of testing in production where shadow deployment of the model against the clinical prediction occurred to determine the performance of the model against the clinician’s prediction. Shadow deployment is where the model generates unused predictions in the background, which tests the effectiveness of the model in a real-life clinical environment without impacting patient experience, an essential step in determining the efficacy of the model in a real-life setting [62]. This is essential for determining whether a MLOps implementation will be safe and useful for operational decision-making in a clinical setting.

Five studies did not incorporate CM and CL due to resource constraints or the elementary stage of their MLOps implementation [34–38]. For example, Granlund et al [37] did not have CM and CL since their model was deployed in a “locked” or “frozen” state as governed by the European Union legislation for medical devices. “Frozen” models, which are not updated postdeployment, are supported by some regulatory bodies to maintain the consistency and safety of the ML models [63,64]. “Frozen” models are beneficial because they provide a stable and predictable performance, which is critical for maintaining trust among health care professionals and patients [65]. However, the downside is that “frozen” models may become outdated as new data and trends emerge, potentially reducing their accuracy over time. Without proper CM and CL, models can degrade in performance, resulting in unreliable predictions which can create more risk for the ML model use case [66,67]. While full MLOps maturity can be preferable with full human oversight, it is not always feasible due to limitations, such as regulations, expertise, and infrastructure. Furthermore, there are risks to full MLOps maturity as well. Due to adversarial attacks on the newly trained ML model, new errors can be introduced by training on new data (eg, a change in how the data was recorded, leading to inaccurate data), and the ML model may undergo catastrophic forgetting [22,24,25]. Hence, there must be a balance between maintaining model stability with the risks introduced by retraining while ensuring periodic updates to capture new insights and evolving data trends. Finally, another important consideration for CM and CL is the decision of the performance metric that will trigger the retraining of the ML model. The choice of the metric is important as well since different ML model metrics measure different aspects of the model performance; for instance, sensitivity focuses on the ability of an ML model to identify true positive cases, and specificity measures the ability of an ML model to identify true negatives.

### Clinical Considerations for MLOps Implementations

Furthermore, an essential consideration for medical MLOps applications discussed by 2 studies [38,43] was the presence of bias in ML models and the incorporation of bias checking within the MLOps workflow pipeline. Bias in ML models can lead to unfair treatment of certain patient groups and hinder the model’s ability to make accurate predictions for individuals of certain backgrounds and underestimate their health care needs [68,69]. Bias can stem from various sources, including nonrepresentative training data, model design choices, and the historical and systemic inequities embedded in the health care system that resulted from differential routine care for different populations [70-72]. To make improvements upon this concern, we must ensure diverse and representative training data and conduct fairness audits. These audits involve evaluating the model’s performance across different groups to identify and mitigate any biases. External validation can also mitigate bias by testing the model’s performance on completely new data, ensuring it can generalize beyond the training environment, which is crucial for reliable clinical use. This process helps identify any hidden biases that were not apparent during internal validation. Furthermore, in cases where external validation may not be feasible, temporal validation can be used where the validation set consists of the data from the same patient population in a future time frame [73]. Finally, testing the ML model in a clinical environment through shadow deployment can help identify potential biases and assess its effectiveness and safety in real-world clinical decision-making.

, because the health care environment itself contains special considerations for patient care, there are notable distinctions between ML deployments in health care and other industries. This is because traditional MLOps principles focus on the technical aspects of operationalizing ML algorithms, such as speed of delivery and management of the data workflow, in addition to the engineering teams working on the MLOps pipeline workflows [12]. In comparison, health care is a conservative environment where there are numerous other considerations surrounding patient ethics, legal implications, clinical workflows, and characteristics of the health care data that will impact how an MLOps workflow will be operated. One key challenge is that current health care data infrastructure is primarily designed for the recording of health service–related events for operational purposes. However, this strategy may not support the technical requirements for MLOps implementation. Thus, health care data infrastructure needs to be adapted for MLOps applications. Furthermore, an essential step of MLOps specific to health care applications would be the inclusion of community stakeholders throughout the entire process of developing the ML tools, especially when engaging community stakeholders enables the development of inclusive ML models that add value to the community and the clinical setting [74,75]. Specifically, it is important for community stakeholders and clinicians to collaborate to develop acceptable standards for features used in the model, model performance, model interpretability, and how to maintain the model long term. Especially when medical systems demand adaptive ML models due to changes in the patient population or clinical environment, as ML model performance will decay over time [76,77]. Achieving full MLOps maturity in health care MLOps systems requires close human oversight. Human oversight must be present for the risk management of the MLOps implementation to ensure that the ML model is still performing to an acceptable standard before the redeployment of the new model [22]. Strategies for ensuring the safety of the newly retrained model could be the shadow deployment of the model or testing models on specific sensitivity population subgroups and consultation with clinicians before a full release. However, there may be instances where full MLOps maturity may not be feasible for clinical environments, as clinical workflows may not have sufficient infrastructure to support the full maturity of the ML pipeline workflow. In these situations, the use case of the clinical models may be important to work with key stakeholders to determine the level of maturity that is acceptable. For example, in clinical use cases where there are minimal shifts in the data distributions, the ML model may be manually updated at lower frequencies.

### Limitations

Five main limitations were identified within this study. First, the quality of the studies varied; hence, it was a challenge to identify every MLOps workflow pipeline step detailed in Table 3 and Multimedia Appendix 3, as each study had a different way of describing the MLOps workflow pipeline steps. Furthermore, the reported details of each MLOps workflow pipeline differed, as certain studies gave details regarding each step while other studies did not discuss certain steps of their pipeline. Hence, it was assumed that if a step in the MLOps workflow pipeline was not discussed, the authors did not complete the step. In addition, there were different descriptions for CM and CL in different studies, so if a described step matched our definition, we would assume that CM and CL were completed. Second, MLOps is poorly defined in the health care realm; there were limited studies in the health care space identified as MLOps studies, so there may be MLOps studies that may not be identified and screened by the protocol. A potential mitigation strategy for the future may be to include MeSH terms within the search strategy. Third, because we only included studies in English, studies in other languages were left out. Fourth, scoping reviews inherently have limitations [78]. While they aim to summarize a broad range of literature, this breadth can sometimes compromise the depth and quality of information on specific topics. Quality assessments of the included studies were not performed as these evaluations are beyond the methodological scope and purpose of scoping reviews [79]. This absence of quality evaluation can hinder the assessment of the strength and reliability of the evidence. Finally, the extracted studies contain limitations to their research design, as health care–specific essential steps for MLOps were not discussed.

### Conclusions

In conclusion, we have examined how MLOps is implemented in health care settings and proposed a 3-stage MLOps maturity framework for health care. Even though certain clinical settings may not have full MLOps maturity, it is important to push for full maturity as ML models can better reflect the dynamic nature of the health care data and the population it serves. Widespread clinical use requires rigorous validation, and CM and CL, and close collaboration with health care providers and regulatory units to ensure these models and the platform that the models are hosted on meet the high standards required for patient care. Current MLOps implementations exist at 3 different maturity stages: low maturity, partial maturity, and full maturity, with thirteen models discussed in the reviewed studies having shown promising results and being on the path to clinical implementation with full MLOps maturity.

## Supplementary material

10.2196/66559 Multimedia Appendix 1 Details of search terms used for Ovid MEDLINE, Scopus, Web of Science, and Embase.

10.2196/66559 Multimedia Appendix 2 Extraction template categories and locations of extracted items.

10.2196/66559 Multimedia Appendix 3 Full descriptions extracted from each study related to Machine Learning Operations pipelines and maturity frameworks.

10.2196/66559 Checklist 1 Preferred Reporting Items for Systematic Reviews and Meta-Analyses extension for Scoping Reviews checklist for Scoping Reviews.

## Abbreviations

CL: continual learning
CM: continuous monitoring
ML: machine learning
MLOps: Machine Learning Operations
PCC: “Population,” “Concept,” and “Context”
PRISM-ScR: Preferred Reporting Items for Systematic Reviews and Meta-Analyses extension for Scoping Reviews
PRISMA: Preferred Reporting Items for Systematic Reviews and Meta-Analyses
