# Supplementary material for: Maturity Framework for Operationalizing Machine Learning Applications in Health Care: Scoping Review
Source: J Med Internet Res. 2025 Sep 19;27:e66559. doi: 10.2196/66559 (PMC12448258; doi:10.2196/66559)
Supplement: Multimedia Appendix 1 [file jmir-v27-e66559-s001.docx]

| Database | Search Terms |
| --- | --- |
| Ovid Medline | 1) DevOp*.mp.  2) exp Machine Learning/  3) ("Artificial intelligence" or "AI" or Bayes* or classifier* or "classification algorithm*" or "computer heuristic*" or "decision tree" or "deep learning" or "back* propagation" or "feature detection" or (Markov adj3 model*) or "learning algorithm*" or "machine learning" or "supervised learning" or "unsupervised learning" or "reinforcement learning" or ((multi?factor* or multi?criteria) adj3 "decision analysis") or "nearest neighbor*" or "neural net*" or "outlier detection" or "pattern recognition" or "random forest" or "support vector machine*" or medical software).mp.  4) 2 or 3  5) 1 and 4  6) ("machine learning operations" or "MLOps" or "artificial intelligence operations" or "mlhops" or "ml-ops" or "clinical MLOps" or "CD4ML" or "Continuous Integration/Continuous Delivery" or "CI/CD" or "dataops" or "ML operations" or "ML-devops" or "machine learning devops" or "modelops" or "model-ops").mp.  7) 5 or 6 |
| Scopus | ( ( TITLE-ABS-KEY ( devops ) AND TITLE-ABS-KEY ( "machine learning" OR "Artificial intelligence" OR "AI" OR bayes* OR classifier* OR "classification algorithm*" OR "computer heuristic*" OR "decision tree" OR "deep learning" OR "back* propagation" OR "feature detection" OR ( markov W/3 model* ) OR "learning algorithm*" OR "machine learning" OR "supervised learning" OR "unsupervised learning" OR "reinforcement learning" OR ( ( multi-factor* OR multifactor OR multi-criteria OR multicriteria ) W/3 "decision analysis" ) OR "nearest neighbor*" OR "neural net*" OR "outlier detection" OR "pattern recognition" OR "random forest" OR "support vector machine*" OR "medical software" ) ) OR TITLE-ABS-KEY ( "machine learning operations" OR mlops OR "artificial intelligence operations" OR mlhops OR ml-ops OR "clinical MLOps" OR cd4ml OR " Continuous Inte-gration/Continuous Delivery" OR "CI/CD" OR "Continuous Integration" OR dataops OR "ML operations" OR "machine learning pipeline" OR "ML-devops" OR "machine learning devops" OR "modelops" OR "model-ops") )  AND ( TITLE-ABS-KEY ( health OR healthcare OR medicine OR medical OR pharmac* OR wellness OR wellbeing OR well-being OR quality-of-life OR qol OR hrqol* OR disease* OR outbreak* OR infectio* OR illness* OR patholog* OR pathogen* OR virus* OR injur* OR disab* OR disorder* OR epidemic* OR pandemic* OR deformit* OR birth-outcome* OR fever* OR symptoms OR cancer* OR diabet* OR asthma* OR heart-attack* OR stroke* OR hypertension OR obes* OR "cardiovascular disease*" OR allerg* OR mortality OR morbidity OR death* OR fatalit* OR stress-level* OR high*-stress* OR psychological-stress* OR mental-stress* OR burnout OR psychiatric OR depression OR anxiety OR psychos* OR stress-disorder* OR ptsd OR trauma* OR suicid* OR hospital* OR clinic* OR primary-care OR medical-care OR emergency-room* OR emergency-department* OR doctor* OR physician* OR general AND practitioner* OR nurs* OR "drug discovery" OR "clinical research*" OR epilep* OR "electronic health record*" OR hemotology OR mri OR disease* OR ehr OR primary-care OR "primary care" OR medical-care OR "medical care" OR emergency-room* OR "emergency room*" OR emergency-department OR "emergency department" )  OR SRCTITLE ( health OR healthcare OR medicine OR medical OR pharmac* OR wellness OR wellbeing OR well-being OR quality-of-life OR qol OR hrqol* OR disease* OR outbreak* OR infectio* OR illness* OR patholog* OR pathogen* OR virus* OR injur* OR disab* OR disorder* OR epidemic* OR pandemic* OR deformit* OR birth-outcome* OR fever* OR symptoms OR cancer* OR diabet* OR asthma* OR heart-attack* OR stroke* OR hypertension OR obes* OR "cardiovascular disease*" OR allerg* OR mortality OR morbidity OR death* OR fatalit* OR stress-level* OR high*-stress* OR psychological-stress* OR mental-stress* OR burnout OR psychiatric OR depression OR anxiety OR psychos* OR stress-disorder* OR ptsd OR trauma* OR suicid* OR hospital* OR clinic* OR primary-care OR medical-care OR emergency-room* OR emergency-department* OR doctor* OR physician* OR general AND practitioner* OR nurs* OR "drug discovery" OR "clinical research*" OR epilep* OR "electronic health record*" OR hemotology OR mri OR disease* OR ehr OR primary-care OR "primary care" OR medical-care OR "medical care" OR emergency-room* OR "emergency room*" OR emergency-department OR "emergency department" ) ) |
| Web of Science | 1: (TS=(Devops)) AND TS=("machine learning" OR "Artificial intelligence" OR "AI" OR bayes* OR classifier* OR "classification algorithm*" OR "computer heuristic*" OR "decision tree" OR "deep learning" OR "back* propagation" OR "feature detection" OR ( markov NEAR/3 model* ) OR "learning algorithm*" OR "machine learning" OR "supervised learning" OR "unsupervised learning" OR "reinforcement learning" OR ( ( multi-factor* OR multifactor or multi-criteria OR multicriteria ) NEAR/3 "decision analysis" ) OR "nearest neighbor*" OR "neural net*" OR "outlier detection" OR "pattern recognition" OR "random forest" OR "support vector machine*") and Preprint Citation Index (Exclude – Database)  2: TS=("machine learning operations" OR mlops OR aiops OR "artificial intelligence operations" OR “dataops” OR “ml-ops” OR “mlhops” OR “mlh-ops” OR "clinical MLOps" OR cd4ml OR " Continuous Inte-gration/Continuous Delivery" OR "CI/CD" OR "Continuous Integration" OR dataops OR "ML operations" OR "machine learning pipeline" OR "ML-devops" OR "machine learning devops") and Preprint Citation Index (Exclude – Database)  3: TS=("machine learning operations" OR mlops OR "artificial intelligence operations" OR “dataops” OR “ml-ops” OR “mlhops” OR “mlh-ops” OR "clinical MLOps" OR cd4ml OR " Continuous Integration/Continuous Delivery" OR "CI/CD" OR dataops OR "ML operations" OR "ML-devops" OR "machine learning devops" OR "modelops" OR "model-ops" )  4: #1 OR #3 and Preprint Citation Index  5: TS=(health or healthcare or medicine or medical or pharmac* or wellness or wellbeing or well-being or quality-of-life or QoL or HRQOL* or disease* or outbreak* or infectio* or illness* or patholog* or pathogen* or virus* or injur* or disab* or disorder* or epidemic* or pandemic* or deformit* or birth-outcome* or fever* or symptoms or cancer* or diabet* or asthma* or heart-attack* or stroke* or hypertension or obes* or "cardiovascular disease*" or allerg* or mortality or morbidity or death* or fatalit* or stress-level* or high*-stress* or psychological-stress* or mental-stress* or burnout or psychiatric or depression or anxiety or psychos* or stress-disorder* or PTSD or trauma* or suicid* or hospital* or clinic* or primary-care or medical-care or emergency-room* or emergency-department* or doctor* or physician* or general practitioner* or nurs*) OR  SO=(health or healthcare or medicine or medical or pharmac* or wellness or wellbeing or well-being or quality-of-life or QoL or HRQOL* or disease* or outbreak* or infectio* or illness* or patholog* or pathogen* or virus* or injur* or disab* or disorder* or epidemic* or pandemic* or deformit* or birth-outcome* or fever* or symptoms or cancer* or diabet* or asthma* or heart-attack* or stroke* or hypertension or obes* or "cardiovascular disease*" or allerg* or mortality or morbidity or death* or fatalit* or stress-level* or high*-stress* or psychological-stress* or mental-stress* or burnout or psychiatric or depression or anxiety or psychos* or stress-disorder* or PTSD or trauma* or suicid* or hospital* or clinic* or primary-care or medical-care or emergency-room* or emergency-department* or doctor* or physician* or general practitioner* or nurs* or ehr or mri or electronic health record* or hemotology)  6: #4 AND #5 and Preprint Citation Index (Exclude – Database) |
| Embase | 1) DevOp*.mp.  2) exp Machine Learning/  3) ("Artificial intelligence" or "AI" or Bayes* or classifier* or "classification algorithm*" or "computer heuristic*" or "decision tree" or "deep learning" or "back* propagation" or "feature detection" or (Markov adj3 model*) or "learning algorithm*" or "machine learning" or "supervised learning" or "unsupervised learning" or "reinforcement learning" or ((multi?factor* or multi?criteria) adj3 "decision analysis") or "nearest neighbor*" or "neural net*" or "outlier detection" or "pattern recognition" or "random forest" or "support vector machine*" or medical software).mp. 603063  4) 2 or 3  5) 1 and 4  6) ("machine learning operations" or "MLOps" or "artificial intelligence operations" or "mlhops" or "ml-ops" or "clinical MLOps" or "CD4ML" or "Continuous Integration/Continuous Delivery" or "CI/CD" or "dataops" or "ML operations" or "ML-devops" or "machine learning devops" or "modelops" or "model-ops").mp.  7) 5 or 6 |
