# Supplementary material for: Maturity Framework for Operationalizing Machine Learning Applications in Health Care: Scoping Review
Source: J Med Internet Res. 2025 Sep 19;27:e66559. doi: 10.2196/66559 (PMC12448258; doi:10.2196/66559)
Supplement: Multimedia Appendix 2 [file jmir-v27-e66559-s002.docx]

| Extraction Template Categories | Location in Manuscript |
| --- | --- |
| Title | Table 2, Figure 2, Supplementary Table 3 |
| Author and Year | Table 1, Table 2, Figure 2, Table 3, Supplementary Table 3 |
| Population/ Disease characteristics | Table 1 |
| Location | Table 1 |
| Aim of Study | Table 1 |
| MLOps Maturity Level | Table 2, Figure 2, Supplementary Table 3 |
| Data Extraction | Table 2, Figure 2, Supplementary Table 3 |
| Data Preparation/Engineering | Table 2, Figure 2, Supplementary Table 3 |
| Model Training | Table 2, Figure 2, Supplementary Table 3 |
| Measured ML Metrics/ Evaluation | Table 2, Figure 2, Supplementary Table 3 |
| Model Validation and test in production | Table 2, Figure 2, Supplementary Table 3 |
| Model Serving and Deployment | Table 2, Figure 2, Supplementary Table 3 |
| Continuous (CM, CL) | Table 2, Figure 2, Supplementary Table 3 |
| Other Considerations | Table 3 |
