# Supplementary material for: Maturity Framework for Operationalizing Machine Learning Applications in Health Care: Scoping Review
Source: J Med Internet Res. 2025 Sep 19;27:e66559. doi: 10.2196/66559 (PMC12448258; doi:10.2196/66559)
Supplement: Multimedia Appendix 3 [file jmir-v27-e66559-s003.docx]

Supplementary Table 3: Full descriptions extracted from each study related to MLOps pipelines and maturity frameworks. In total, 19 studies were reviewed and organized across three stages—data preparation, model development, and model operationalization—and three levels of maturity: Low Maturity, Partial Maturity, and Full Maturity.

|  | | | Data Preparation | | | Model Development | | Model Operationalization | | |
| --- | --- | --- | --- | --- | --- | --- | --- | --- | --- | --- |
| Title | Year of Publication and author | MLOps Maturity Level | Data Extraction | Data Engineering | Model Training | | Measured ML Metrics/ Evaluation | Model Validation and test in production | Model Serving and Deployment | Continuous (CM, CL) |
| Towards Regulatory-Compliant MLOps: Oravizio’s Journey from a Machine Learning Experiment to a Deployed Certified Medical Product | Granlund 2021 | Low Maturity | Over 30,000 patient records was used for the development of Oravizio. | A data lake was built to consolidate the data from different sources. The preprocessed data included 750 variables relating to patient demographic and other clinical measures. To determine the top variables for building the model, LASSO, Ridge Regression, and Elastic net, and expert clinical guidance were used for feature selection. | Because the model must be transparent, logistic regression, decision tree methods, gradient boosting, and Cox survival models were used to develop the ML models. | | AUC and ROC was used to evaluate the best fitting model. | The model was validated using the test data. The model was not validated in the final production environment, where the model was deployed. Internal validation was completed. | The model was deployed in a “locked state” where the ability for the model to make the prediction was not improved in production for regulatory purposes. | N/A |
| Deepepil: Towards an Epileptologist-Friendly AI Enabled Seizure Classification Cloud System based on Deep Learning Analysis of 3D videos | Karacsony 2021 | Low Maturity | The Neurokinect system captured and streamed video data for seizure patients per day. The data set for the MLOps framework contains 500 epileptic seizures from 100 patients. | These epileptologists are responsible for providing the clinical labels for the epileptic seizures video data. Furthermore, patient data, classifications of seizure type, and other specifications for the seizures are provided by the clinicians. | Machine learning models were developed by the data scientists of the team. The ML models were developed using Tensorflow, Keras, and PyTorch. | | N/A | N/A | The user-interface for the ML model was developed in React, a library used for building user interfaces, to provide a user-friendly application for the clinicians. The back-end is based in Python, a programming language. To establish connection between the user-interface and machine learning model, flask app is used, which accepts user requests from the clinician interface to feed information into the machine learning models. PostgreSQL was used to manage the patient data. | N/A |
| Machine Learning Smart System for Parkinson Disease Classification Using the Voice as a Biomarker | Tougui 2022 | Low Maturity | The study used the mPower Public Research Portal database. The smartphone voice recordings and demographic information was used from the data base. | To extract the voice recording data from raw audio files, Python and SQL was used with the Synapse REST API. The demographic data was used to identify Parkinson’s patients and healthy controls. Extractions on 123 features of significance for predicting Parkinson’s was completed on the audio recordings using the Surfboard library.  The data was scaled and imputed. LASSO or ElasticNet was used to determine feature importance for feature selection. | The machine learning models used were a gradient boosting classifier (GBC) with a LASSO penalty, and GBC pipeline with ElasticNet. | | Accuracy, recall, and the F1-score was used to assess the performance of the model. | Internal validation was conducted. | Model was served in a user-friendly desk top application where users can manage the audio data and the model performance. | N/A |
| AutoPrognosis 2.0: Democratizing diagnostic  and prognostic modeling in healthcare with  automated machine learning | Imrie 2023 | Low Maturity | The dataset for the proof of concept ML application is from the UK Biobank where the 502,467 individuals were chosen for the diabetes risk prediction. | The set of 109 features were chosen based on clinical expertise and literature review. | Machine learning models (ADABoost, ExtraTree, TabNet, and Neural Network) were developed and compared to the performance of traditional statistical models such as the Cox proportional hazard model. | | Metrics used to assess the performance of the ML models include Area under the receiver-operating curve (AUROC), Brier score, and the C-index | Internal validation was performed. Comparison of the predicted risk scores made by the ML to classic clinical risk scores was also performed. | The best performing machine learning model was deployed on a web application. | N/A |
| Accelerating pharmaceutical R&D with a user-friendly AI system  for histopathology image analysis | Lutnick 2023 | Low Maturity | Researchers processed histology image data for use in the AI pipeline. | Features from the segmented histology images can be stored by the platform. Automated annotation of image segments can be performed by the platform. | A weakly supervised ML algorithm with a transformer architecture was used to support histology image analysis and predictions. | | N/A | An internal validation was performed. | The best performing models are delivered on a web application for end users. | N/A |
| DataOps Lifecycle with a Case Study in Healthcare | Bahaa 2023 | Partial Maturity | University of California Irving (UCI) Machine Learning Heart Disease dataset was chosen for this application. A subset of 14 features were chosen from the dataset relating to demographic information and cardiac-related physiological measures. | The feature engineering phase was done using Python 3 and the Jupyter Notebook IDE. The data set was loaded manually, and cleaned. | Eight ML models were trained and tested. Examples of models trained and tested were logic regression, naïve bayes, and support vector machines. Two separate models were developed, with the top four, and all 13 features. | | Accuracy, F1, sensitivity, and Specificity were considered for all eight models. | N/A | Recommendations are related to the implementing APIs to deliver to model, and specifications for the delivery of any data insights. | CM ONLY |
| Fast Healthcare Interoperability Resources for Inpatient Deterioration Detection with Time-Series Vital Signs: Design and Implementation Study | Tseng 2022 | Full Maturity | ETL (extract, transform, load) was completed from the health information system database into the FHIR (Fast Healthcare Interoperability Resources) interface. | Data engineering was completed to derive 16 features (e.g., gender, heart rate, patient_ID) needed for the prediction of In-Hospital Cardiac Arrest (IHCA). Vital signs features include: respiratory rate , heart rate, systolic blood pressure, diastolic  blood pressure, and body  temperature. | A long short term memory network was trained using the vital signs data to predict the risk of IHCA. | | ML metrics were obtained. | N/A | A dashboard was created containing early warning signals to alert medical professions of abnormal vital signs in patients, and performance from machine learning models. HTTP GET was used to obtain features to train and test the predictive model for patient risk of IHCA. | CM, CL |
| Implementation of Machine Learning Pipelines for Clinical Practice: Development and Validation Study | Kanbar 2022 | Full Maturity | For the epileptic surgical candidacy predictions, Oracle SQL queries were performed to extract patient data. Patient data was labeled as surgery seizure-free, and unknown outcome.  For the automated clinical trial eligibility screener (ACTES), real time RestfulAPI was used to extract real time patient data in regards to demographics, medical orders, and patient notes. | For the preprocessing of the data, features were extracted from the patient notes. The Java Natural Language Processing pipeline LingPipe was used to extract and preprocess patient notes for both clinical applications.  The patient notes for both clinical applications were hand annotated by the physicians. | Support Vector machines, Naïve Bayes, and random forest prediction models were develop for predicting surgical candidacy scores in epilepsy patients. | | The epilepsy intervention system performed at a sensitivity of 71% and positive predictive value of 77%, which is just as good as board certified neurologists in identifying candidates eligible for surgery.  For the clinical trial recommender system, research coordinators spent 12.9% less time on screening.  Systems were evaluated for biases influenced by demographics such as race. It was determined that demographic variables such as race and gender were not a source of bias for the system. | Shadow deployment was implemented for one year to determine the accuracy of the ML system compared to the clinical environment. | An interactive web platform was built to inform physicians on patients who are classified as potential surgical candidates.  ACTES was integrated into the clinical research coordinators’ workflow to support  real-time patient screening. The patients recommended for screening was refreshed every 10 minutes for real time updates to research coordinators. | CM, CL |
| Digital Twin in Healthcare Through the Eyes of the Vitruvian Man | Kleftakis 2022 | Full Maturity | Each set of patient data was stored in an SQL database, which will be cleaned and assessed for data reliability. | There was a “feature store” that contains the features that are used for training and testing the ML model. The raw data is cleaned for data imputation, and consistent data quality. | The best performing model (e.g., Linear Regression, Decision Tree, and Naïve Bayes) was selected using HyperOpt, a hyperparameter tuning library. | | For each model, performance statistics were collected to determine if a new model needs to be trained. | For a new model to be put into production, it needs to surpass the accuracy of the previous model.  Internal validation. | The model was served to the patient and stored in a model registry to track the models that are used. | CL,CM |
| CyclOps: Cyclical development towards Operationalizing ML models for health | Krishnan 2022 | Full Maturity | Extract, transform, load was performed on the data set into a clean structured format. Data from the General Internal Medicine Unit from the Greater Toronto Area was used. | Cleaning, aggregation, imputation, and normalization of the data was completed. | Various ML models like LSTM (long short term memory) were trained and tested for the different applications. | | ML metrics such as AUROC were measured. | N/A | N/A | CM, CL |
| Automatic Enhancement of  Deep Neural Networks for Diagnosis of COVID-19 Cases with  X-ray Images Using MLOps | Kundu 2023 | Full Maturity | The data set used in this study employed 200 samples of COVID-19 and 200 samples of healthy control chest X-rays. | The samples were equally divided into training and testing dataset. For the experimental setting, all photos were scaled to 224 224 pixels in size. | The authors trained the model using pre-trained weights from the CheXNet implementation, a convolutional neural network. | | Precision, recall, F1-Score, and ROC was determined for predicting the predicting the presence of COVID-19 using chest X-rays. | N/A | N/A | CM, CL |
| Melatect: A Machine Learning Approach for Identifying Malignant Melanoma in Skin Growths | Meel 2022 | Full Maturity | The data set employed in this study consists of 54,052 images of skin lesions. The skin lesion data was extracted from International Skin Imaging Collaboration (ISIC) data set. | The images of the skin lesions were rotated, resized, and Gaussian noise was added. | A pre-trained VGG-16 convolutional neural network was employed. | | F1, AUC, and accuracy was used to assess the model. There are ~94:6 true to false positives ratio AUC of 95.23, and the accuracy is 96.6%. | The model was validated to ensure that it does not contain bias, and achieves the desired performance.  Internal and external validation with clinical trials. | The ML model was deployed in an IOS app using Flask, a Python library that deploys ML models. | CM, CL |
| A clinical site workload prediction model with machine learning lifecycle | Mirza 2023 | Full Maturity | Data extraction was completed from 150,000 historical raw records. The outcome is defined as the amount of time completing source data verification. | The data was scaled, and the minimum and maximum values for the training set was determined by subject matter experts. | A LSTM (long short term memory) model was used. | | Evaluation metrics for the ML model include Mean Absolute Error ,Root Mean Square Error (RMSE) , Median Absolute Percentage Error (MAPE). | Internal validation was conducted.  Comparison to clinical standards was also completed. | Model was served using Domino, an open source platform used for deploying and maintaining ML models. | CM, CL |
| A Graphical Toolkit for Longitudinal Dataset Maintenance and Predictive Model Training in Health Care | Bai 2022 | Full Maturity | The health informatician transformed the clinical data into SQL tables and defined the outcome and predictor variables. | The data was prepared to be imported into an SQL database. Patient privacy was ensured by removing all 18 protected health information (PHI) identifiers. | Users of the platform can import ML models of their choice into the MLOps platform. | | The models are assessed via F1 Score, Accuracy, Sensitivity, Specificity, and AUC on the dashboard. | Internal and external validation was completed. | The best performing models are served via a RESTful API. The clinical ML model was created as a graphical user interface to improve usability. | CM, CL |
| Fuzzy Enhanced Kidney Tumor Detection:  Integrating Machine Learning Operations for a  Fusion of Twin Transferable Network and  Weighted Ensemble Machine Learning Classifier | Ghosh 2025 | Full Maturity | A data set comprising of 5,977 healthy and 2283 kidney tumor images was used to develop the machine learning prediction model and feature selection methods. | An ensemble feature vector was created using PT-CNN (pre-trained convolutional neural networks). | An ensemble model was developed comprised of SVM, RF, KNN, LR, and NB. | | The measured ML metrics were accuracy, Precision, Recall, and F1 Score, specificity, kappa, Matthews correlation coefficient, Classification  Success Index (CSI), and Good Detection rate were used to measure the performance of the ensemble model. | Internal validation was completed on a pre-existing data set. | The model is containerized using Docker and deployed on a Google Cloud platform. | CM, CL |
| Digital Twin for Continual Learning in Location Based Services | Lombardo 2024 | Full Maturity | Simulated data was constructed through the real time recording of the locations of the healthcare staff throughout the hospital. | Feature scaling, outlier detection, and dimensionality reduction were applied to the data. | An anomaly detection task on the trajectory of hospital staff was constructed using a long short term memory (LSTM) neural network. | | Accuracy Precision, Recall and F-1 score was measured for the trajectories of the physicians, patients,  and supporting staff. | An internal validation was performed. | Model is served on a digital interface. | CM, CL |
| Machine-learning operations streamlined clinical workflows of DNA  methylation-based CNS tumor classification | Markowitz 2024 | Full Maturity | Data was procured from DNA methylation arrays for cancer diagnosis collected on site at the hospital, in addition to public databases such as the Gene Expression Omnibus database. | Bioinformatics preprocessing of the genetic data was completed. | K-nearest neighbour (KNN) and random forest models were used to make predictions | | AUC, specificity, accuracy, concordance rates with clinical diagnosis are used to select the model used for deployment. | Internal validation was performed and external validation was performed in a clinical setting. | N/A | CM, CL |
| Applying Transfer Learning on 3D Brain Images and an MLOps Study for Deployment | Mathew 2023 | Full Maturity | 3D MRI brain images were used for the development of the MLOps system. | Features were extracted using the Residual group attention network (ResGANet) kernels. | The pipeline utilized the Residual group attention network (ResGANet) for tumor prediction. | | Accuracy, dice-coefficient and final loss were used to assess the performance of the final model. | Internal validation was conducted. | Best performing model is packaged and served on a flask app hosted on a Google Cloud platform. | CM, CL |
| Resilience-aware MLOps for AI-based medical diagnostic system | Moskalenko 2024 | Full Maturity | MedMNIST datasets were used for the testing of the MLOps system. | N/A | The pipeline utilized the ResNet-18 model. | | Accuracy was used to assess the performance of the model. | Internal validation was conducted. | N/A | CM, CL |
